# Assessment of Physical Activity among Adolescent Girls in the West of Iran: Status, Limitations and Solution

**Published:** 2018-06

**Authors:** Aso SALAHYAN, Sakineh RAKHSHANDEROU, Mohtasham GHAFFARI, Yadollah MEHRABI

**Affiliations:** 1. Environmental and Occupational Hazards Control Research Center, Dept. of Public Health, School of Health, Shahid Beheshti University of Medical Sciences, Tehran, Iran; 2. Environmental and Occupational Hazards Control Research Center, Dept. of Epidemiology, School of Health, Shahid Beheshti of Medical Sciences, Tehran, Iran

## Dear Editor-in-Chief

Insufficient physical activity (IPA) is one of main risk factors for many diseases, such as hypertension, cardiovascular disease, diabetes, osteoporosis, cancers, depression, anxiety, obesity, and others (1). There is general agreement in scientific community that an improved level of physical activity has contributed strongly to a decrease in mortality worldwide (2). In spite of the growing scientific knowledge on the benefits of physical activity for health, levels of physical activity seem to be declining among adolescents, especially in poor communication (3).

The objective of this study was to describe the levels of physical activity among adolescent girls student (13–15 yr) in Sanandaj City, West of Iran. We attempted to explore the association between IPA and demographic data and parental variables and finally change behavior based on education intervention to promoting this public health issue among adolescent girls. A further aim was to test the validity and reliability of the questionnaire used.

In this descriptive-analytical study, 219 girl students of first grade of junior high school were selected completely randomly from four schools in different district of Sanandaj City

Most of studies use questionnaires to collect data about physical activity due to financial and logistical constraints (1), therefore, we similarly used questionnaire for data collection in the present research.

Data collection instrument in this study is a 2-section questionnaire including 9 items was related to demographic information and general information and 19 items was related to measurement of physical activity of students. To measure the physical activity, Self-report Questionnaire of Garcia was used. Based on current physical activity guidelines for adolescents, IPA was defined as less than 60 min per day of moderate- to vigorous-intensity physical activity exercise (4).

Data analysis by using SPSS (Chicago, IL, USA) showed that 43.3% of students preferred physical activity (60 min or more per day) with moderate to vigorous-intensity throughout the day, 25.6% moderate physical activity (less than 60 min) and about 30.6% of students have IPA (Table 1). No significant correlation was found between level of physical activity in students and parents occupation status, and education level of parents (Table 2).

**Table 1:** Level of physical activity among adolescent girls in Sanandaj City (behavior)

***Level***	***Definition***	***N (%)***
Desired	60 min or more per day with moderate to vigorous-intensity	95 (43.3)
Moderate	40 up to 59 min per day	56 (25.6)
Insufficient	less than 40 min per day	67 (30.6)

**Table 2:** Physical activity of Adolescents based on demographic variables

***Variable***		***N (%)***	***Mean ± SD***	***P-value***
Age group (yr)	13	86 (39.3)	36.49 ± 25.95	
	14	87 (39.7)	38.50 ± 25.77	
	15	46 (21.0)	37.0 ± 30.58	0.883
Educational status (Father)	Illiterate	64 (29.2)	34.45 ± 29.88	
	Primary school	69 (31.5)	41.42 ± 25.67	
	Secondary school	58 (26.5)	36.90 ± 24.92	0.476
	Diploma and Collage	28 (12.8)	35.35 ± 26.06	
Educational status (Mother)	Illiterate	33 (15.0)	35.0 ± 28.01	
	Primary school	68 (31.0)	34.30 ± 27.04	
	Secondary school	54 (24.6)	40.33 ± 26.66	0.534
	Diploma and Collage	64 (29.2)	39.49 ± 26.20	
Job (Father)	Employed	60 (27.4)	37.60 ± 26.68	
	Self-employment	110 (50.0)	38.61 ± 25.64	
	Worker	49 (22.2)	34.47 ± 29.71	0.668
Job (Mother)	House wife	31 (14.0)	45.89 ± 25.91	0.994
	Practitioner	188 (86.0)	36.01 ± 26.75	

Regular physical activity is an important behavior for health enhancement and disease prevention (4). In actuality, sufficient physical activity has been associated with numerous health benefits in adolescents (5).

Physical activity is a priority for health promotion policies (6). Data of adolescents were recruited and showed that most adolescents have IPA (7), and IPA tend to increase during adolescence (7). Similarly, many adolescent girls do IPA to attain and maintain an appropriate health. In addition, conduct education intervention leads to increase in the average scores of behavior and eventually helps to improved physical activity among adolescent girls in Sanandaj city. These results are consistent with the observations reported (8). Some studies have pointed to the proximity to recreational public park is one of the most important predictors of physical activity (9). This is in agree with our finding, lake of neighborhood facilities for physical activity was also cited by the most adolescent girls as barriers to physical activity practice in Sanandaj City.

Finally, the rate of IPA is low among adolescents’ girl and necessary the development of interventions for increasing physical activity levels. Moreover, health officers should, therefore, design training programs focusing on improvement IPA.

## Financial support

This work was a part of the M.Sc. thesis of Aso Salahyian, supported financially by Shahid Beheshti University of Medical Sciences (Grant No. 6640).
